# Assessment of Mint, Basil, and Lavender Essential Oil Vapor-Phase in Antifungal Protection and Lemon Fruit Quality

**DOI:** 10.3390/molecules25081831

**Published:** 2020-04-16

**Authors:** Renata M. Sumalan, Raufdzhon Kuganov, Diana Obistioiu, Iuliana Popescu, Isidora Radulov, Ersilia Alexa, Monica Negrea, Amonullo F. Salimzoda, Radu L. Sumalan, Ileana Cocan

**Affiliations:** 1 Faculty of Horticulture and Forestry, Banat’s University of Agricultural Sciences and Veterinary Medicine “King Michael I of Romania” from Timisoara, 119 C. Aradului, 300645 Timisoara, Romania; 2 Department of Food Storing and Processing Technology, Faculty of Agribusiness, Tajik Agrarian University named Shirinsho Shotemur, 146 Rudaki, Dushanbe 73400, Tajikistan; a.faizullozoda@mail.ru; 3 Interdisciplinary Research Platform, Banat’s University of Agricultural Sciences and Veterinary Medicine “King Michael I of Romania” from Timisoara, 300645 Timisoara, Romania; diana.obistioiu@yahoo.com; 4 Faculty of Agriculture, Banat’s University of Agricultural Sciences and Veterinary Medicine “King Michael I of Romania” from Timisoara, 300645 Timisoara, Romania; isidoraradulov@yahoo.com; 5 Faculty of Food Engineering, Banat’s University of Agricultural Sciences and Veterinary Medicine “King Michael I of Romania” from Timisoara, 300645 Timisoara, Romania; alexa.ersilia@yahoo.ro (E.A.); negrea_monica2000@yahoo.com (M.N.); negreaileana@yahoo.com (I.C.)

**Keywords:** IC50, fungicide effect, GC/MS analysis, *Penicillium digitatum*, ascorbic acid

## Abstract

There is an increasing interest in developing natural methods to replace the current chemicals used for maintaining postharvest quality of citrus fruits. The essential oil antifungal activity of mint (MEO), basil (BEO), and lavender (LEO) acting as the vapor-phases was tested against *Penicillium digitatum*. The minimum doses with fungistatic and fungicidal effect, in vitro, acting as the vapor-phases, were set up. The minimum fungicidal dose was 300 μL for BEO and 350 μL LEO, while for MEO only minimal dose with fungistatic effect was reached. The IC50 values were calculated and used (*v*/*v*) for testing preservation of lemon fruits, in close space enriched in vapor oil. For this purpose, the following two independent in vivo experiments were carried out: experiment 1, inoculated lemons with *P. digitatum* stored without chemical treatments 7 days, at 22 ± 2 °C, at two concentrations (C1—IC50 equivalent; C2—half of C1); and experiment 2, the non-inoculated lemons kept under the same conditions and concentrations of EO vapor served to evaluate the lemon quality properties. The results showed that antifungal protective effect was provided in the order of LEO-C1 > BEO-C1 > MEO-C1 > BEO-C2 > MEO-C2 > LEO-C2. The quality indicators like weight loss, pH, and firmness were not negatively influenced.

## 1. Introduction

Nowadays, the major concern in postharvest research is keeping the phytonutrients, which assures the nutritional value of fruits and vegetables while minimizing the losses during storage [1]. The fungal decay of lemon fruits (*Citrus limon*) is the main cause of microbiological spoilage during postharvest, leading to high economic declines due to the high water content and to the wounds that often form as a result of harvesting and transportation. The main fungus genus responsible for lemon depreciation is *Penicillium*, among which *Penicillium italicum* is responsible for blue rot, and *Penicillium digitatum* causes green rot [2,3,4,5]. 

Because consumers are more and more concerned about the use of synthetic preservatives, the exploitation of natural compounds has been intensively researched in recent years [6,7,8,9,10]. Innovations in preserving horticultural commodities can be achieved through three directions: (1) introduction of biocontrol agents, such as yeasts and bacteria [11,12,13]; (2) use of plant essential oils (EOs) extracted from thyme, mint, lemongrass, lemon balm, oregano, or savory [5,14,15,16]; and (3) by physical methods like sulphur dioxide fumigation, use of ozone, or mixed techniques [7,17,18].

The antifungal activity of EOs has been known and used for centuries, and nowadays efforts to promote natural compounds in post-harvest control of horticultural products have led to an increased interest in their possible applications [19,20,21,22,23]. A particular problem regarding the use of essential oils is related to the decrease in concentration of bioactive compounds due to evaporation [24].

The volatilization properties of essential oils was the idea behind this study. Therefore, it would be particularly interesting to know what amount of essential oil is required to achieve the fungistatic and fungicidal action of the EO vapor-phase against fruit fungal depreciation, and what essential oil is more economically efficient, given their high costs [24]. Considering this background, the aim of this work was to study the effectiveness of the vapor-phases of the essential oils (EOs) of *Mentha piperita* (MEO), basil (*Ocimum basilicum*, BEO), and lavender (*Lavandula angustifolia*, LEO) in lemon preservation for antifungal protection against *P. digitatum* and the effect on fruits quality indicators. 

Our research could help to extend the method of storage of freshly cut vegetables or fruit in a modified atmosphere based on the use of natural volatile compounds. To the best of our knowledge, this is the first time that essential oils in the vapor-phase were tested as a preservation method to prevent fungal lemon degradation.

## 2. Results

### 2.1. Chemical Composition of Essential Oils by GC/MS Analysis 

The MEO, BEO, and LEO composition was determined by the GC/MS method and 23 different components of MEO, 12 of BEO, and 23 of LEO were identified (Table 1). Regarding the extraction of the essential oils, the highest yield was obtained for the dry mass of lavender with 4.85%, followed by the mint with 3.22%, and basil with 0.28%. 

About 99.99% of the total constituents were detected in MEO. Of them *p*-menthan-3-one had the highest percent (31.00%), followed by menthol (25.19%), 1,3,12-nonadecatriene (9.76%), eucalyptol (7.44%), and carvone (6.72%). The oxygenated monoterpene compounds predominated in a proportion of 81.28%; sesquiterpene oxygenated compounds were not detected.

BEO had the lowest diversity of compounds compared with MEO and LEO. Only 12 compounds were identified (99.68% proportion), the majority being monoterpene oxygenated compounds (97.02%). The predominant compound was estragole (49.94%), followed by linalool (41.49%) and eucalyptol (3.46%). Other compounds were found in concentrations lower than 1%. LEO showed a chemical composition similar to that of MEO, 23 compounds being identified of which two represented the major constituents linalool (31.44%) and linalyl acetate (31.78%). The next constituents in lower proportion were 4-terpineol (8.43%), caryophyllene (5.39%), and lavandulol (5.24%). LEO was the singular EO that contained the sesquiterpene oxygenated compound, caryophyllene oxide, in a low proportion (0.35%).

### 2.2. In Vitro Assay of MEO, BEO, and LEO Vapors for Antifungal Performances

**Figure 1:**
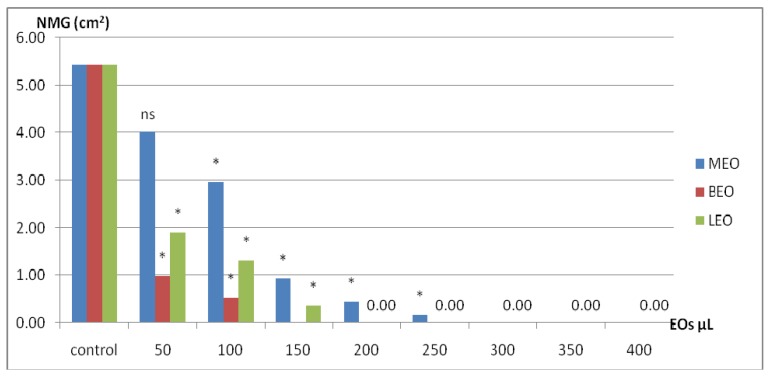
The NMG (new mycelium growth) of *P. digitatum* in EO vapor-phase modified atmospheres in the in vitro assay (cm^2^). MEO, mint essential oil; BEO, basil essential oil; LEO, lavender essential oil; * with statistical differences compared to control for *p* ≤ 0.05, *n* = 4; ns, without statistical significance.

The presence of essential oil vapor led to inhibition of mycelial growth of *P. digitatum* in a dose-dependent manner. The EO dose used in the experiment provided the vapor-enrichment of Petri dish atmosphere for 50 cm^3^ of air according to Formula (2). From the first dose of added essential oils (50 μL) we observed decreases of new mycelium growth, but only for BEO and LEO were the differences statistically significant (Figure 1). 

The mycelial growth of the fungus became null at 150 μL BEO, 200 μL LEO, and 300 μL MEO. Thus, these doses can be attributed to the minimum inhibitory dose which provides a fungistatic effect (MFsD), as seen in Table 2. After passing the mycelium on CYA medium without added EOs, we found that the mycelium restarted the growth after 4 days of incubation. Where the fungal growth was not resumed, the dose of the EO used was considered the minimum dose with fungicidal effect (MFdD).

Therefore, by transferring the *P. digitatum* mycelia disks, it was possible to estimate the two different doses for each type of EO, as seen in Table 2. Thus, MFsD for BEO was reached at 150 μL (0.3%), and BEO 300 μL (0.6%) had a fungicidal effect. LEO, in the presence of linalool and linalyl acetate (together totaling 63.22%) achieved the MFsD at 200 μL (0.4%) and MFdD at 350 μL (0.7%), while for MEO only MFsD was reached at 300 μL.

### 2.3. Experiment 1: In Vivo Assay of Inoculated Lemon Fruits Stored with EO Vapor-Phase

**Figure 2:**
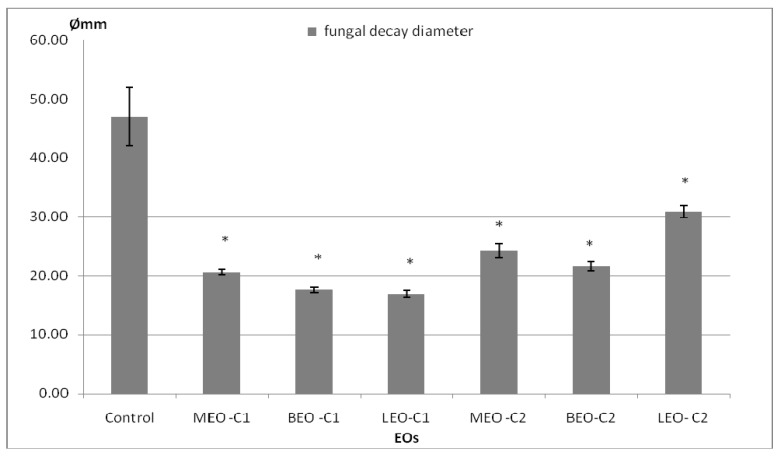
Results of lemon depreciation inoculated with *P. digitatum* stored seven days in the modified atmosphere with EO vapor. Data represent the diameter (mm) affected by the fungal growth with or without reproductive structure presented as mean ± SD. Vertical bars represent standard deviations (*n* = 8); * with statistical differences compared to control at *p* < 0.05; MEO-C1 = 183 μL·L^−1^, BEO-C1 = 46 μL·L^−1^, LEO-C1 = 86 μL·L^−1^, MEO–C2 = 91.5 μL·L^−1^, BEO-C2 = 23.0 μL·L^−1^, LEO-C2 = 43.0 μL·L^−1^.

After 7 days of lemon storage in atmosphere enriched with essential oil vapor, the mycelium growth of *P. digitatum* was measured. The non-treated control lemons recorded the largest average diameter of 47 mm Ø followed in order by LEO-C2 ˃ MEO-C2 ˃ BEO-C2 ˃ MEO-C1 ˃ BEO-C1 and finally LEO-C1 with an average of fungal diameter of 16.88 mm (Figure 2). 

EO results tested by fumigation of artificially inoculated lemon fruits were compared and classified according to the *t*-test for independent samples. It was observed that between BEO-C1 and LEO-C1 there were no statistically significant differences (Table 3). The same aspect was noted for BEO-C2 and MEO-C2. The lowest effect was recorded for LEO-C2, ranking in second place after the control for the antifungal performance.

### 2.4. Experiment 2: Physiological and Biochemical Indicators of Lemon Quality

Loss of fruit firmness, assessed by the penetration power of lemon fruits, was influenced by type and concentration of EO vapor, as seen in Table 4. In this regard, the treatment LEO-C2 (65.1 N) and MEO–C1 (59.0 N) showed significant differences compared with the control (46.6 N). In contrast, treatments with BEO, at both concentrations (C1 and C2), did not provided significant differences versus the control.

Regarding the weight loss after 7 days of storage in fumigated EO vapor, the smallest decrease was noted for the fruits kept in LEO-C1 treatment. For the rest, the differences were statistically significant (values expressed as a percentage from initial control). Analyzing the pH value, it was found that keeping the lemons in EO vapor had no influence; there were no significant differences compared with the control.

**Figure 3:**
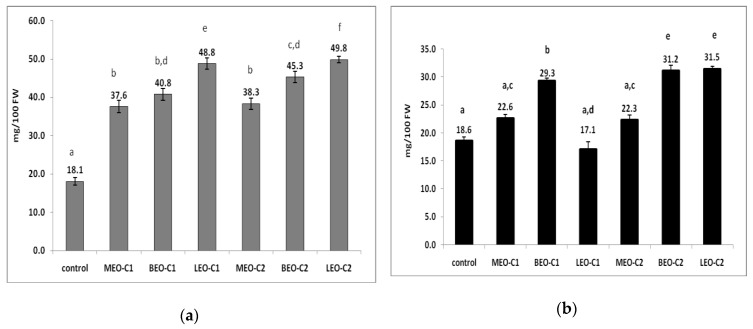
Levels of ascorbic acid in the peel (**a**) and pulp (**b**) of lemons after 7 days of storage in enriched air-space with EO vapor. Data represent means ± SD (mg AsA·100 g FW). Different letters on top of column indicate differences for *p* < 0.05, *t*-test. Vertical bars represent standard deviations for *n* = 10; MEO-C1 = 183 μL·L^−1^, BEO-C1 = 46 μL·L^−1^, LEO-C1 = 86 μL·L^−1^, MEO–C2 = 91.5 μL·L^−1^, BEO-C2 = 23.0 μL·L^−1^, LEO-C2 = 43.0 μL·L^−1^.

Citrus fruits are widely recognized for rich content in ascorbic acid (AsA). As can be seen from Figure 3a, the AsA content increased in lemon peel in all EO vapor treatments during 7 days of lemon fumigation. The smallest differences compared to the control were noticed for treatments MEO-C1, MEO-C2, and BEO-C1, respectively, differences which proved to be without statistical significance. The highest AsA was determined for LEO-C2. In addition, it can be noted that the C2 concentration of the EO treatments led to a higher AsA content accumulated in lemon peels, compared to C1 concentration. This observation was only valid for BEO and LEO.

In the lemon pulp, the amount of AsA after 7 days of storage in the atmosphere enriched by EOs was higher in all treatments except one, as seen in Figure 3b. For LEO-C1 the amount of AsA determined (17.1 gr 100^−1^g FW) did not ensure significant differences compared to the control (18.6 gr 100^−1^ g FW). In contrast, LEO-C2 provided the highest amount of AsA in the lemon peel of 31.5 gr 100^−1^ g FW. Similar to AsA content in lemon peel the C2 EO concentration caused a greater amount of AsA accumulation than C1 concentration.

## 3. Discussion

Essential oils differ from other oils due to the presence of volatile aromatic compounds that plants synthesize in stressful moments [21]. Mostly, the antimicrobial effect of essential oils is due to the predominant compounds of essential oil; based on this observation different chemotypes have been distinguished within aromatic species [26,27,28].

Obviously, the antifungal effect of EO against *P. digitatum* is mainly due to the presence of monoterpene oxygenated compounds [29]. Within this group, the following compounds represent the majority, as seen in Table 1: menthol in MEO, linalool and methyl chavicol in BEO, and linalool in LEO. This is in accordance with the findings of other researchers; they have shown that linalool and terpinen 4-ol acts against *Aspergillus fumigatus* through vapors and not through agar diffusion contact action [30].

Considering the definition of the minimum inhibitory dose (MID) as the lowest dose that determines 100% inhibition of fungal growth, similar with MIC for testing EO in liquid [29,31], we must consider one important aspect, namely the particularity of fungus to regenerate. Therefore, it appears imperative to define two different aspects: (1) the minimum dose leading to a temporary inhibition of fungal growth, in which case the fungistatic effect was achieved (MFsD); and (2) the minimum dose necessary for fungal irreversible inhibition, when the hyphae do not regenerate, and the fungicidal effect is achieved (MFdD). To be noted, in the case of MEO the establishment of MFdD, the minimal fungicidal dose, was not successful, and future investigations are needed to determine the fungicidal effect of MEO for doses greater than 400 μL (0.8%, *v*/*v*). Taking into account the mycelia growth in the presence of EO vapor valid for 50 cm^3^ of Petri dish headspace, the IC50 values for each EO were established using the polynomial equation (Supplementary Materials Figure S1). Accordingly, the doses of EO that provided 50% inhibition of *P. digitatum* growth were 91.49 μL (0.183%, *v*/*v*) for MEO, 23.28 μL (0.046%, *v*/*v*) for BEO, and 43.39 μL (0.086%, *v*/*v*) for LEO. The good antifungal performance of BEO can be due to the synergic action of the two majority compounds like estragole and linalool.

Keeping lemons in the EO enriched atmosphere has the advantage that fruits are not in direct contact with oils because they act on fungi due to its volatilization properties. From what is known so far, only MEO among the EOs tested is recommended for fumigation-based methods due to volatilization properties [32]. However, the action of the EO vapor-phase has proven to be more effective against *Trichophyton mentagrophytes* and *Aspergillus fumigatus* than has been shown in agar essential oil diffusion assays [33]. Similarly, in MEO, BEO, and LEO performed by vapor, the amplitude of EO antifungal effects occurred in a dose-dependent manner (Figure 1). Thus, the presence of MEO vapors assures the antifungal effect due to the menthol, whereas the p-menthan-3-one compound (similar to menthone) is known to have no antifungal action [34,35]. Existence of other compounds, such as eucalyptol (7.44%), carvone (6.72%), and D-limonene (3.06%) recognized for their antimicrobial activity, could interact together or synergic with menthol, thus enhancing the antifungal action. The mode of action of vapor compounds from EO is different, being capable of disrupting the hyphal cells or fungal spore membrane, inhibiting the sporulation, and finally affecting the growth of food spoilage fungi [35]. Identically, the antifungal potential of BEO is due to the presence of the alcoholic compounds linalool, 41.49%, and estragole, 49.94%, (similar to methyl chavicol), which also has proven antifungal activity [28]. The presence of estragole in a high proportion, above the value of linalool, includes the basil plant used in the study into estragole chemotype. Generally, it is widely accepted that the chemical composition of BEO exhibits fluctuations for each component, mainly due to the climatic conditions but influenced by genotype as well. 

Similarly, for LEO, linalool represents a major compound of about 31.44% and together with linalyl acetate (31.78%) ensures the antifungal effect of LEO. Additionally, if the ratio value of linalyl acetate-linalool is above one, lavender oil is considered to be of high quality [36]. In the case of the lavender essential oil used in our experiment, the ratio was 1.011, which attests the high quality of the oil. Ranking the EO vapor treatments by *t*-test analysis revealed that the same intensity of the antifungal effect could be reached with different amounts of EOs (Table 3). Evidently, this can be explained by the different proportion of antifungal compounds contained in EOs. The most effective EOs for in vivo antifungal preservation of lemons was significant by LEO-C1 with 86 µLL^−1^, and BEO-C1 with 46 µL·L^−1^, respectively. Both essential oils contain linalool as a major compound, but we must make it clear that lemon peel has antifungal compounds such as D-limonene and citral that can potentiate the antifungal effect of the linalool. This argument is in line with Bakkali et al., who affirm that the minority compounds have an important role in the biological effect of EOs by facilitating the penetration of the microbial cell wall or membranes [37]. The antifungal protective effect for lemons was provided in order of LEO-C1 > BEO-C1 > MEO-C1 > BEO-C2 > MEO-C2 > LEO-C2.

Physiological quality indicators like fruit firmness depend on the EO dose used. Firmness is one of the most important indicators used to assess the quality of many fruits. Loss of fruit firmness during storage shows that there are water losses and metabolic changes in fruits [23]. Different polysaccharide, protein, or carboxymethyl cellulose (CMC) coating techniques or edible coatings with chitosan bilayers do not form effective water-vapor barriers [38]. Thus, essential oil vapor-phase treatment is proving to be more efficient during the post-harvest manipulation of fruits. Until now research on the application of cinnamon and eucalypt EO vapor treatments (50 ppm) have been reported with positive effects on maintaining firmness for tomato crop and cherry tomatoes [23]. Additionally, Jiang et al. noted that in shiitake mushrooms after fumigation with cloves, thymus, and cinnamaldehyde EO vapors for 20 days at 4 °C storage, the firmness, texture, and resistance to microorganism attack were augmented. As a result, the mushrooms were better preserved compared to the control sample during storage, but at the same time a higher concentration of EO can have an undesirable effect [39].

Regarding the pH value, it was found that keeping the lemons in EO vapor did not influence the pH values. The result is in agreement with previous research that showed that citrus fruits treated with wax and citral did not change their pH values after 5 days of storage [40].

AsA is one of the most frequently studied and powerful antioxidants. Fruit cells have the ability to protect themselves from oxidative stress by producing low molecular mass antioxidant molecules; in this category ascorbic acid and phenolic compounds are included [41]. Until now, EO was used especially in coating mixtures with chitosan or wax [10]. It appears that certain compounds from the structure of essential oils, such as ethylene, act on lemons in oxidative stress, triggering the enzymatic pathway for the synthesis of AsA molecules [10,39]. Therefore, AsA accumulation in large quantities, especially in the lemon peel, confirms this. However, not all EO vapor can cause the stimulant effect. MEO vapors in both concentrations did not induce ascorbic acid synthesis. In our experience, BEO and LEO vapor have proven elicitor properties. Additionally, other researchers applied thyme EO vapor in peach treatment and reported increased activities of defense-related enzymes and total phenolic content [42]. 

The results obtained regarding AsA increases, in lemon pulp and peel, in the presence of EO vapor, are in line with recent studies on treatment with compounds such as methyl jasmonate, nitric oxide, salicylic acid, and essential oil that have proven to elicit and stimulate the inductive-defensive system and extend the shelf life of fruits and vegetables [43,44,45]. 

## 4. Materials and Methods 

### 4.1. Plant Materials, EO Extraction, and Gas Chromatography/Mass Spectrometry Assessment

The research was performed between April and August 2018 in the Interdisciplinary Research Platform of Banat University of Agricultural Sciences and Veterinary Medicine (Romania). Aerial parts of plants, including mint, lavender, and basil, were collected from experimental fields of the Aromatic Plant Department, Faculty of Agriculture Timisoara. The samples were harvested in full flowering stage, in the months of June and July, on sunny days with moderate water deficit when the essential oil content was the highest [21]. Plant samples were cleaned, dried in the shade, milled, and then stored in the dark until use. An amount of 300 g of a homogenous sample was used for steam distillation for 2.0 h, using Clevenger-type equipment for essential oil extraction according to the European Pharmacopeia [46]. The obtained oils were stored at 2–4 °C until GC/MS analysis. The extraction yields of MEO, BEO, and LEO were calculated using the following formula:

Yield (%) = (amount of EO (g)/amount of dry plant (g)) × 100
(1)

The chemical characterization of EO was done using a gas-chromatograph equipment with a Shimadzu QP 2010Plus, Columbia, SC, USA mass spectrometer (GC/MS) with an AT WAX capillary column (characteristics 30 m × 0.32 mm × 1 µm). Helium was used as the carrier gas with a flow rate of 1 mL/min with a column pressure of 42 kPa. Component separation was achieved under the following program: 40 °C for 1 min a rate of 5 °C/min to 210 °C for 5 min. Injector and ion source temperatures were 250 °C and 220 °C, respectively. The injection volume was 1 µL of a hexane solution of EO with a 1:50 split ratio. The NIST 5 Wiley 275 libraries database was used to identify volatile compounds through previously calculation of linear retention index (LRI) [25].

### 4.2. Antifungal Efficacy Assessment: In Vitro Assay

The fungal isolate, denoted Pd_0318_L, from Microbial Culture Collection, Agricultural Microbiology Department, Faculty of Horticulture and Forestry Timisoara, was used in both in vitro and in vivo assessments. The fungus was previously isolated from spoiled lemons on MEA (malt-extract-agar, Sigma-Aldrich, Chemie, Madrid, Spain) and was identified, by morphological and cultural characteristics according to Pitt and Hocking, as *Penicillium digitatum* [47]. To test the antifungal efficacy of EO vapor, two circular plugs per Petri dish (8 mm Ø) were picked up from the edge of a 4 day-old *P. digitatum* mycelium and were transferred on CYA medium (Czapek-yeast-agar, Sigma-Aldrich). The amount of medium distributed was exactly 20 mL on each Petri dish (Ø = 100 mm). After mycelia plug inoculation (two per Petri dish) to ensure an atmosphere with essential oil vapor, in the lid of the Petri dish a sterile filter paper was placed on which the dose of essential oil was added. The variants were as follows: 0.0 (control), 50, 100, 150, 200, 250, 300, 350, 400 µL for MEO, BEO, and LEO, respectively. To prevent the escape of volatile compounds, the Petri dishes were sealed with adhesive tape. The volume of vapor enriched air in each Petri dish was calculated according to the following equation:

V_HS_ = VPD − V_M_ (2)

where V_HS_ is the headspace of the Petri dish with EO vapor; V_PD_ is the volume of the Petri dish (70 cm^3^); and V_M_ is the volume of media in the Petri dish (20 cm^3^).

This means that for each Petri dish, 50 cm^3^ of air was enriched in EO vapor, resulting in the following concentrations (%, *v*/*v*): 0.1, 0.2, 0.3, 0.4, 0.5, 0.6, 0.7, and 0.8, respectively, valid for each EO used in the experiment. The research was performed twice.

After 5 days, two perpendicular diameters of mycelia were measured, and their average (AD) was calculated. The formula for calculating the new mycelium grown surfaces (NMG) is

NMG = ((AD^2^ ∗ 3.14/4) − AFI)/100 (3)

where NMG is the new mycelium growth surface (cm^2^); AD is the average of two perpendicular diameters of the fungal colony (mm); and AFI is the circular plug area of fungal inoculums (50.24 mm^2^).

For assessment of fungistatic (MFsD) or fungicidal (MFdD) doses, the fungal discs from treatment samples with no growth recorded were re-inoculated into fresh CYA medium, and after 3 days the fungus revival was checked. The NMG values were then used to determine IC50 (EO concentration with 50% mycelia inhibition). 

### 4.3. Experiment 1—Antifungal Protection of Lemons, In Vivo Assay 

Lemon (*Citrus limon*, Verna cv) fruits without chemical treatments were used for the evaluation of antifungal protection in the atmosphere enriched in the essential oil vapor-phase. Fourteen desiccant containers of 2 L volume were used; in each of them, 4 lemons were tested as replicates. In total, 56 lemons fruit were washed first in a 9:1 hypochlorite (NaOCl) solution and then rinsed three times in sterile water. After drying in a sterile niche, the lemons were wounded in the peel at the equatorial zone with a cork borer (0.8 cm diameter and 0.5 cm deep); two wounds were produced on each lemon. The wounds were inoculated with 100 µL spore suspension of *P. digitatum* in saline water (0.65%) containing 10^5^ spores·mL^−1^. For each type of essential oil, two doses were tested, in two repetitions each. Thus, EO vapor fumigation treatments were denoted as MEO-C1, MEO-C2, BEO-C1, BEO-C2, LEO-C1, LEO-C2, and the control without EOs. Each treatment was done twice. C1 represents dose of EOs corresponding to the IC50 value, and C2 is the half of it, as seen in Table 5. 

The essential oil was added on the filter paper at the bottom of the desiccant container along with a Petri glass with 10 mL distilled water for maintaining 90% relative humidity (RH). Four artificially inoculated lemons were placed on the grid. The desiccant containers were hermetically sealed with grease and stored at 24 ± 2 °C in the dark for 7 days. The in vivo experiment was performed twice. 

### 4.4. Experiment 2: Physiological and Biochemical Indicators of Lemon Fruit Quality, In Vivo Assay

Forty lemons of the Maglina variety, purchased from the supermarket (conventional culture from Greece), were divided into 8 groups (five lemons per group serving as repetitions). For the initial determination of the indicators, namely firmness, the content of ascorbic acid in peel and pulp, and the pH value, a group of five lemons was used. For the firmness index of the lemons (*n* = 5), a digital penetrometer FR 5120 (tips 8 mm, manufacturer LUTRON ELECTRONIC ENTERPRISE, Taiwan) was used. The rest of the lemons were used to monitor the quality indicators after a 7-day storage period in an atmosphere enriched with EO vapor. The lemon preservation was tested in hermetically desiccant containers (2 L volume each) inside of which 5 lemons (taken as repetitions) were placed. The required volume of each EO, in order to ensure the concentrations C1 and C2 according to Table 5, was added on a filter paper placed on the bottom of the container. Each lemon fruit was weighed, recorded, and labelled prior to storage in EO vapor-enriched air-space. After 7 days of storage in the dark at 24 ± 2 °C, 90% RH, the lemons were weighed again, and the differences were presented as percent of weight loss compared to the initial weight using Formula (4).

Weight loss (%) = IW − FW ∗ 100/IW
(4)

where IW is the initial weight; and FW is the final weight.

The pH value was determined using an automatic WTW inoLab pH 720 (Weilheim, Wissenschaftlich-Technische Werkstätten GmbH, Germany). The contents of ascorbic acid (AsA) from peel and lemon pulp were determined with the Tillman method using 2,6-dichloroindophenol (Tillman’s reagent, TR) from an average of 5 lemons per treatment [48]. Briefly, after weighing and determining lemon firmness, the peel was scratched until the white lemon skin was exposed. An amount of 4 g was weighed from an average sample to obtain the extract. Therefore, for each gram, 10 mL of 70% ethanol (ethanol 96%, Merck KGaA, Darmstadt, Germany) was added, and the sample was shaken for 120 min at room temperature using a GFL 3005 Analogue Orbital Shaker (Gesellschaft für Labortechnik GMBH, Burgwedel, Germany). Extracts were filtered using Whatman membrane filters (nylon, 0.45 µm, 30 mm diameter; Sigma-Aldrich; Merck, Germany). AsA content was estimated titrimetrically using 5 mL of extract diluted with 5 mL of oxalic acid (CAS Number 144-62-7, Merck, Germany), adding 1 mL acetaldehyde (6.9 g L^−1^; CAS Number 75-07-0, Merck, Germany) and then titrated with 1 mM solution 2,6-dichloroindophenol sodium salt hydrate (Merck, Germany) to pink color. The experiment was performed twice.

### 4.5. Statistical Analysis

The results of both in vivo and in vitro assays, regarding the potential of EO treatments, were presented as mean ± standard deviation (SD). ANOVA analyses were applied for data obtained from new mycelial growth area and diameters of fungal growth on lemons preserved with EO vapor treatment. The IC50 for each EO was established by the polynomial equation; previous data were log-transformed. Additionally, *t*-test analysis was performed for ranking the EO treatments acting as vapor in the storage of lemons, and for AsA accumulation in lemon peel and pulp. Data were processed with Statistica 10 (StatSoft, USA).

## 5. Conclusions

Due to the volatilization properties, essential oils represent a natural method with high potential for use in preserving or storing organic fruits and vegetables. EO utilized in the vapor-phase could be effective against fungal deteriorations. Our data suggest that integration of EO treatments in the vapor-phase could be an alternative to enhance the health-promoting phytochemicals in lemons.

The presence of EO in the vapor-phase stimulates the activity of the defense system in fruits by increasing the ascorbic acid content responsible for protecting against the oxidative stress.

Our study clearly indicated that the vapor activity of BEO contributed considerably to the inhibitory diameter of *P. digitatum*, and suggested, from an economic point of view, that BEO has better efficiency than LEO and MEO since it needs the smallest quantity of EO in the lemon antifungal preservation for the same time period.

For the future, research is needed using higher doses of mint essential oil or research under controlled conditions, taking into account lower temperature, humidity control, and the possibility of airflow to determine the maximum potential that each EO can provide. 

## Figures and Tables

**Table 1:** The chemical composition of essential oils, GC/MS analysis (% from total compounds).

Compounds	Type	LRIc/LRIr	MEO	BEO	LEO
α-Pinene	MH	1021/1015	0.62	-	-
β-Pinene	MH	1106/1096	0.89	0.40	-
Thujene	MH	1118/1122	0.50	-	-
β-Myrcene	MH	1158/1164	0.31	-	0.36
*p*-Mentha-2,4(8)-diene	MH	1176/1180	0.23	-	-
d-Limonene	MH	1196/1193	3.06	0.39	0.62
Eucalyptol	MO	1204/1209	7.44	3.46	1.48
*trans*-β-Ocimene	MH	1228/1230	-	-	4.75
Gamma Terpinene	MH	1241/1242	0.43	-	-
*cis*-β-Ocimene	MH	1245/1250	-	0.62	4.02
*p*-Cymol	MH	1263/1264	-	-	0.35
*o*-Cymol	MH	1265/1268	-	0.31	-
6-Methyl hept-5-en-2-one	MO	1325/1325	-	0.30	-
Octen-1-ol acetate	MO	1364/1365	-	-	1.20
*p*-Menthan-3-one	MO	1457/1458	31.00	-	-
*cis*-Linaloloxide	MO	1460/1463	-	-	0.14
Menthofurane	MO	1474/1477	1.38	-	-
d-Menthone	MO	1484/1486	3.19	-	-
Camphor	MO	1507/1518	-	-	0.26
Linalool	MO	1533/1537	0.39	41.49	31.44
Linalyl acetate	MO	1541/1543	0.94	-	31.78
Menthyl acetate	MO	1552/1551	2.20	-	-
*p*-Menth-8-en-3-one	MO	1562/1561	0.19	-	-
1-Terpineol	MO	1565/1562	-	-	0.17
Alfa Santalene	SH	1571/1574	-	-	0.56
4-Terpineol	MO	1593/1592	-	--	8.43
Caryophyllene	SH	1598/1599	2.35	-	5.39
Cyclohexanone,5 methyl-2-(1 methylethyliden)	MO	1635/1633	2.07	-	-
Estragole/Methyl chavicol	MO	1652/1650	-	49.94	-
Beta Farnesene	SH	1653/1652	-	-	1.35
8-*p*-Menthen-2-ol	MO	1655/1656	0.24	-	
Cryptone	MO	1658/1661	-	-	0.47
Cis Citral	MO	1664/1668	-	0.66	-
Alfa Terpineol	MO	1694/1697	-	-	0.13
Germacrene D	SH	1708/1708	0.57	-	0.45
Trans Citral	MO	1712/1714	-	0.80	-
3-Carvomenthenone	MO	1713/1710	0.32	-	-
Carvone	MO	1719/1718	6.72	-	-
Alfa Bisabolene	SH	1734/1736	-	0.94	-
3-Isopropylbenzaldehyde	MO	1765/1765	-	-	0.13
Menthol	MO	1801/1788	25.19	-	-
Anethole	MO	1807/1817	-	-	0.69
Lavandulol	MO	1879/1879	-	-	5.24
Caryophyllene oxide	SO	1998/1989	-	-	0.35
Eugenol	MO	2198/2186	-	0.37	-
1,3,12-Nonadecatriene	SH	2405/2400	9.76	-	-
Total (%)			99.99	99.68	99.76
from which	MH		6.06	1.72	10.10
	MO		81.28	97.02	81.56
	SH		12.65	0.94	7.75
	SO		-	-	0.35

LRIc, calculated linear retention index; LRIr, referred linear retention index [25]; MH, monoterpene hydrocarbonates; MO, monoterpene oxygenated; SH, sesquiterpene hydrocarbonates; SO, sesquiterpene oxygenated.

**Table 2:** Values of fungistatic and fungicidal doses of essential oils used as the vapor-phases in the in vitro study.

EO Treatment	Effect	EO Doses (µL)							
		50	100	150	200	250	300	350	400
MEO	MFsD ^a^	+	+	+	+	+	-	-	-
	MFdD ^b^						+	+	+
BEO	MFsD ^a^	+	+	-	-	-	-	-	-
	MFdD ^b^			+	+	+	-	-	-
LEO	MFsD ^a^	+	+	+	-	-	-	-	-
	MFdD ^b^				+	+	+	-	-

^a^ minimum dose with fungistatic effect; ^b^ minimum dose with fungicidal effect.

**Table 3:** Ranking the EO treatments acting as vapor in the storage of lemons by *t*-test analysis.

Rank	Treatment	Mean *	LEO C1	BEO C1	MEO C1	BEO C2	MEO C2	LEO C2	Control
1	Control	47.0 ^a^	30.1	29.4	26.4	25.4	22.8	16.1	
2	LEO-C2	30.9 ^b^	14.0	13.3	10.3	9.3	6.6		
3	MEO-C2	24.3 ^c^	7.4	6.6	3.6	2.6			
4	BEO-C2	21.6 ^d^	4.8	4.0	1.0				
5	MEO-C1	20.6 ^d^	3.8	3.0					
6	BEO-C1	17.6 ^e^	0.8						
7	LEO-C1	16.9 ^e^							

* mean (*n* = 8) with different superscript letters indicating that the differences are statistically significant for α = 0.05; C1—concentration corresponding to IC50, C2—half of C1 according to Table 1 (MEO-C1 = 183 μL·L^−1^, BEO-C1 = 46 μL·L^−1^, LEO-C1 = 86 μL·L^−1^, MEO–C2 = 91.5 μL·L^−1^, BEO-C2 = 23.0 μL·L^−1^, LEO-C2 = 43.0 μL·L^−1^).

**Table 4:** Effects of essential oils of mint (MEO), basil (BEO), and lavender (LEO) acting as vapor on firmness (N), weight loss (%), and pH recorded in lemon fruits after storage for 7 days.

Treatment	Firmness ^a^ (N)	Weight Loss ^b^ (%)	pH ^b^
Initial control	45.0 ± 9.1 ^A^	-	2.57 ± 0.01
Control	46.6 ± 8.8 ^A^	0.46 ± 0.1	2.56 ± 0.2 ns
MEO-C1	59.0 ± 15.2 ^B^	0.44 ± 0.12 *	2.66 ± 0.12 ns
BEO-C1	45.9 ± 10.8 ^A^	0.51 ± 0.32 *	2.63 ± 0.03 ns
LEO-C1	53.6 ± 15 ^A,C^	0.33 ± 0.13 ns	2.72 ± 0.06 ns
MEO-C2	51.2 ± 19.5 ^A,C^	0.47 ± 0.09 *	2.64 ± 0.01 ns
BEO-C2	48.4 ± 9.5 ^A^	0.42 ± 0.07 *	2.67 ± 0.00 ns
LEO-C2	65.1 ± 7.3 ^B^	0.35 ± 0.11 *	2.69 ± 0.00 ns

^a^ means (*n* = 10) followed by the same letter do not differ significantly at *p* = 0.05; ^b^ means for *n* = 5; * significant difference according to control; ns without significance; C1-concentration corresponding to IC50; C2—half of C1; MEO-C1 = 183 μL·L^−1^, BEO-C1 = 46 μL·L^−1^, LEO-C1 = 86 μL·L^−1^, MEO–C2 = 91.5 μL·L^−1^, BEO-C2 = 23.0 μL·L^−1^, LEO-C2 = 43.0 μL·L^−1.^

**Table 5:** Doses and concentrations of EOs used for the in vivo study of lemon preservation in vapor-phase modified atmosphere.

EO Doses	IC50 In Vitro *		C1 In Vivo **		C2 In Vivo ***	
	EO Doses µL	EO µL L^−1^ Air Space	EO Doses µL	EO µL L^−1^ Air Space	EO Doses µL	EO µL L^−1^ Air Space
MEO	91.49	183.0	3660	183	1830	91.5
BEO	23.28	46.0	932	46	466	23.0
LEO	43.39	86.0	1736	86	868	43.0

MEO, mint essential oil; BEO, basil essential oil; LEO, lavender essential oil; * IC50 values valid for 50 cm^3^ of Petri dish head space; **C1 concentration of EO corresponding to the IC50 value, *** C2 concentration of EO corresponding to half of IC50 value valid for 2 L air from container space.

## Supplementary Materials

Figure S1: The IC50 values determined by polynomial equation for MEO (a), BEO (b), and LEO (c).
