# Supplementary material for: Assessment of Mint, Basil, and Lavender Essential Oil Vapor-Phase in Antifungal Protection and Lemon Fruit Quality
Source: Molecules. 2020 Apr 16;25(8):1831. doi: 10.3390/molecules25081831 (PMC7221751; doi:10.3390/molecules25081831)
Supplement: Supplementary file 1 [file molecules-25-01831-s001.pdf]

Supplementary material

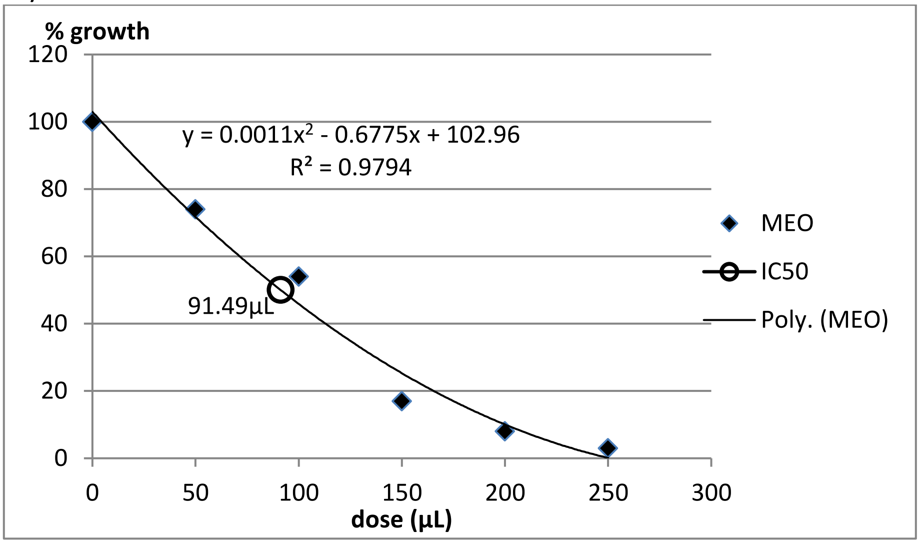

(**a**)

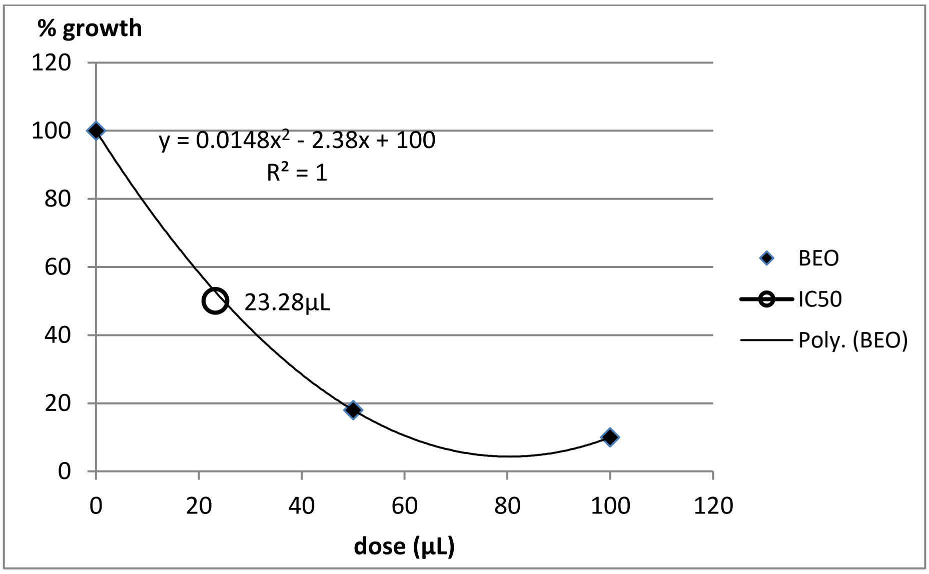

(**b**)

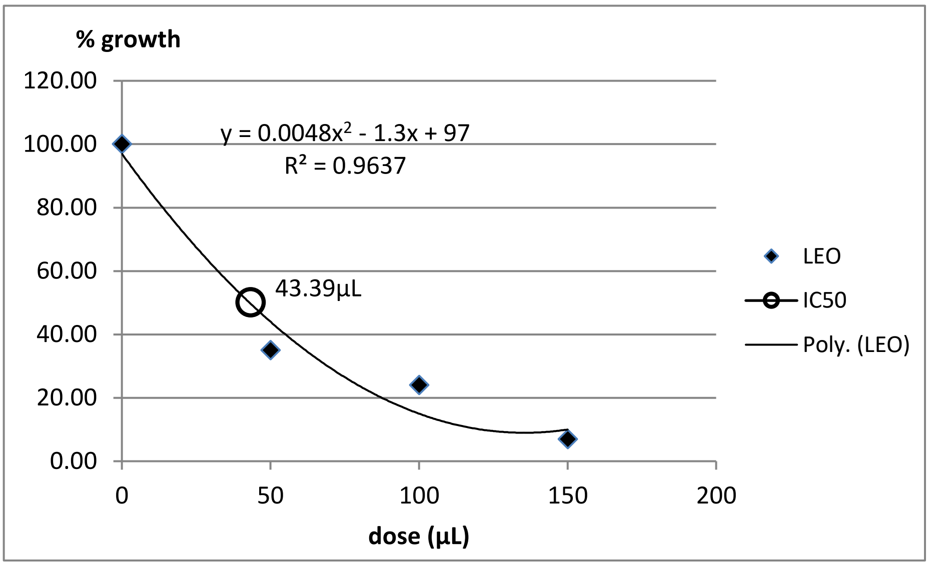

(**c**)

Figure 1S. The IC50 values determined by polynomial ecuation for MEO (a), BEO (b), and LEO (c)
